# Building financial management capacity for community ownership of development initiatives in rural Zambia

**DOI:** 10.1002/hpm.2810

**Published:** 2019-05-23

**Authors:** Viviane I.R. Sakanga, Parker S. Chastain, Kathleen L. McGlasson, Jeanette L. Kaiser, Misheck Bwalya, Melvin Mwansa, Kaluba Mataka, David Kalaba, Nancy A. Scott, Taryn Vian

**Affiliations:** ^1^ Department of Programs Amref Health Africa in Zambia Lusaka Zambia; ^2^ Technology Exchange Lab Cambridge Massachussets USA; ^3^ Biostatistics and Epidemiology Data Coordinating Center Boston University School of Public Health Boston MA USA; ^4^ Department of Global Health Boston University School of Public Health Boston MA USA; ^5^ Department of Research Right to Care Zambia Lusaka Zambia; ^6^ Department of Research Monitoring and Evaluation, Society for Family Health Lusaka Zambia; ^7^ Department of Production Akros, Inc. Lusaka Zambia; ^8^ Department of Finance and Administration Eastern and Southern African Management Institute Arusha Tanzania; ^9^ School of Nursing and Health Professions University of San Francisco San Francisco CA USA

**Keywords:** capacity building, community ownership, financial management, sustainability, training

## Abstract

**Background:**

Building financial management capacity is increasingly important in low‐ and middle‐income countries to help communities take ownership of development activities. Yet, many community members lack financial knowledge and skills.

**Methods:**

We designed and conducted financial management trainings for 83 members from 10 community groups in rural Zambia. We conducted pre‐training and post‐training tests and elicited participant feedback. We conducted 28 in‐depth interviews over 18 months and reviewed financial records to assess practical application of skills.

**Results:**

The training significantly improved knowledge of financial concepts, especially among participants with secondary education. Participants appreciated exercises to contextualize financial concepts within daily life and liked opportunities to learn from peers in small groups. Language barriers were a particular challenge. After trainings, sites successfully adhered to the principles of financial management, discussing the benefits they experienced from practicing accountability, transparency, and accurate recordkeeping.

**Conclusion:**

Financial management trainings need to be tailored to the background and education level of participants. Trainings should relate financial concepts to more tangible applications and provide time for active learning. On‐site mentorship should be considered for a considerable time. This training approach could be used in similar settings to improve community oversight of resources intended to strengthen developmental initiatives.

## 1 BACKGROUND

Domestic resource mobilization is gaining increasing importance in light of the Sustainable Development Goals (SDGs), particularly for community development initiatives in resource limited settings. Community ownership and capacity building are essential principles underlying the SDGs, critical to sustaining any development project in the long term.^1^, ^2^ Domestic resource mobilization at the local level requires financial systems, which include basic procedures for financial control, clear financial roles and lines of accountability, and knowledgeable individuals to keep accurate records and make informed financial decisions.^3^, ^4^, ^5^, ^6^ At the community level, such financial systems must be simple and easy to use: They must be appropriate to the local context while adhering as much as possible to international best practices,^6^, ^7^ and they must respect existing power structures and the skill levels of individuals.^8^

To build sufficient capacity among local communities to manage sustainable development interventions in resource‐limited settings, training of community members in financial management is needed. A key challenge in financial management training is how to best bridge the education‐practice divide, that is, how to ensure that participants have the knowledge, skills, and self‐efficacy to implement in their communities what they have learned during a training.^9^ Prior research has shown that training programs need to integrate practical experiences to develop the skills needed for job‐related responsibilities, such as problem‐solving.^9^, ^10^, ^11^ In addition, training in ways that promote a collaborative and supportive team environment is more conducive to learning for a wide variety of education levels.^12^

As part of a process evaluation of a community‐owned and managed maternity waiting homes (MWH) intervention, we attempted to build the financial management capacity of 10 community groups in four districts of rural Zambia. In collaboration with government,^13^, ^14^ local communities adopted MWHs, residential facilities located next to a health center, to help increase access to emergency obstetric and neonatal care services for the most remote women.^15^, ^16^, ^17^ Historically, the sustainability of MWHs after the termination of external funding has been a challenge in many countries, in part because of insufficient focus in the design of programs on local ownership, daily management, future maintenance, and sources of long‐term, local funding.^18^, ^19^, ^20^, ^21^, ^22^ As such, from inception, our project sought to construct and equip MWHs adapted to local needs and to build capacity for the operational and financial sustainability of the homes through (a) governance committees (GCs), community‐derived decision‐making bodies that oversee the daily operations, financial well‐being, and long‐term missions of the homes and (b) income generating activities, community‐based businesses whose profits would be used to support the MWHs.^13^, ^14^, ^23^ We developed and refined a simplified financial management system, grounded in international best practices^7^ and tailored for the rural communities in which the GCs were operating.

Through financial management trainings on financial terminology, procedures, and controls, we sought to improve the knowledge, skills, and confidence of GC members to manage their selected income generating activity and the financial health of the MWHs. This study describes the trainings conducted, reports pretest and posttest data on knowledge of financial concepts as well as participant feedback on the trainings and qualitatively assesses how well the GCs enacted what they learned over 18 months of posttraining follow‐up. Lastly, we describe how a similar approach could be applied to other community‐level development interventions in resource‐limited settings.

## 2 METHODS

### 2.1 Study setting

This project worked with the communities surrounding 10 rural health centers in Choma, Kalomo, and Pemba Districts of Southern Province, and Nyimba District of Eastern Province, Zambia. The rural health centers were geographically dispersed and were situated between 10 and 135 km from their district urban centers. While some sites appeared geographically close to one another, the roads and transport options between the sites were challenging, often isolating the community groups involved in the intervention.

According to the most recent Zambian Demographic and Health Survey, approximately 70% of the rural population of Zambia has either no formal education or only some primary education, with the representative population from these provinces having completed a median of approximately 3.5 years of formal education, slightly higher among males than females and higher in Southern compared with Eastern Province.^24^ The populations of Nyimba District speak Chinsenga and/or Chinyanja, which are closely related, while the majority of study districts in Southern Province speak Chitonga, with many in the population also knowing Chinyanja.^24^

Embedded within an MWH intervention, a full description of which is available elsewhere,^23^ a project implementation team provided technical assistance in the startup of operations for 10 new MWHs, which included facilitating the establishment of a GC at each site. The GCs are made up of about 10 local stakeholders that include traditional leadership (headmen), local government council members, faith‐based community members, health center staff, neighborhood health committee members, community health workers, other well‐respected members of the community, and women of reproductive age. The members were selected by the community at large by the traditional headmen or by the health center staff in consultation with the local district medical office. The GCs self‐selected their members to participate in one or more of the subcommittees that focused on supervising the (a) MWH, (b) income generating activity, and (c) finances.

The GCs are responsible for overseeing the daily operations and long‐term future of the MWHs, which requires the GCs to raise funds, manage assets, pay operating expenses, and save toward future repairs or maintenance needs and reinvestment. Each GC selected the income generating activity for their site from a menu of pre‐determined options based on local stakeholder input: a goat rearing business, a grinding mill operation, and an agro‐dealership, which sells agricultural inputs. GCs were advised of the projected operating costs for each option and were tasked with conducting a local market analysis and developing a business plan before implementation began. Income generating activity officers were hired at each site to run the businesses on a daily basis.

Project staff trained in accounting worked with the GC leadership and consulted with relevant stakeholders, including nongovernmental organizations and the Ministry of Community Development to develop the MWH financial systems, which included clear roles and lines of accountability; procedures for handling, depositing, withdrawing, and spending money; and record books for documentation of revenue, expenses, and banking. A visual example of the financial system has been provided in Data S1.

### 2.2 Intervention description

The project aimed to improve the capacity of the GC members to manage the finances of the income generating activities and contribute proceeds to the community‐owned MWH. We developed a financial management training that taught basic financial management concepts, such as terminology and budgeting, use of the project‐designed books for record keeping, and processes and procedures for handling of finances specific to the MWH project. The implementation and outcomes of this financial management training are assessed in the following sections.

#### 2.2.1 Participants and trainers

Relevant GC members from each site were invited to participate in the financial management training, including the GC chairperson, treasurer, health center staff representative, income generating activity officer(s), the MWH officer (who runs the MWH daily), and one to two additional members at large of the committee's choosing for a total of seven to nine participants per site. During the course of the trainings, we went through an internal process improvement, adopting a longer training based on participant feedback and trainer observations. The first training (a pilot) lasted 2 days, and the second training (the roll out) was 4 days long. The trainers included the project's Director of Finance and Administration as well as local Zambian project staff members who were part of the implementation support team that either developed or reviewed the financial training manual. All trainers were fluent in English and the local dialect of Chinyanja. Some were also fluent in the additional dialects of participants, including Chitonga. The overall training objectives and participant expectations are included in Table 1 below.

**Table 1:** Financial management training objectives and participant expectations^a^

Training Objectives		Participant Expectations	
1	Relay the purpose of financial management and why we should care about it	1	Know about financial management
2	Define common financial management terms	2	Know how to keep money
3	Describe the different types of revenue and expenses of the MWH	3	Know how to run a business
4	Enter data into a cashbook	4	Know how to make profit in a business
5	Describe the process for banking activities	5	Know how to record money
6	Summarize transactions in a financial report	6	Get a financial management training certificate
7	Interpret a financial report and use the information for decision making		

Abbreviation: MWH, maternity waiting home.

a Training objectives were set by the project in advance, while participant expectations were solicited from participants on the first day of training.

#### 2.2.2 Two‐day training

The first (pilot) training was conducted in early 2017 with the community members from four sites, representing at least one of each type of income generating activity (goat rearing, grinding mill, and agrodealership). Twenty‐eight individuals (seven from each site) participated in the training. The 2‐day training relied mainly on lecturing with some exercises and role play. Participants were introduced to the basic principles of financial management, financial transactions, and the cashbook (one of the financial record books) on day one. The second day consisted of review of financial procedures and recordkeeping. The lectures were conducted in both English and Chinyanja using the training materials that were prepared in English. Written handouts were available only in English.

#### 2.2.3 Four‐day training

The 4‐day training, conducted in mid‐2017, was attended by 54 participants (average of nine individuals from each of the six sites). Based on participants' feedback and the trainers' observation of the challenges faced by the participants during the initial training, the second training was increased to 4 days and allocated more time for role play and exercises that related to the participants' daily interactions (for example, reviewing revenue and expenses for a household), group discussions, task group presentations, and peer‐to‐peer explanations. As with the 2‐day training, the 4‐day training lectures were conducted in both English and Chinyanja, spoken by most participants. Participants fluent in English assisted the training team by translating some of the financial terms into the local dialect (Chitonga) of some participants. For example, terms like *financial management, transaction, capital, expenditure, procurement*, and *reimbursement* needed to be translated, which often involved a group discussion to ensure the terms were fully understood by all participants. Participant translators also assisted the group in translating to other group members during the interactive group exercises. The trainers adapted the instructions to the pace of the slowest person in the group and paired participants to create a peer‐to‐peer learning environment throughout the training period.

The first day was dedicated to reviewing the financial procedures manual, while day two focused on introducing the basic concepts and terminologies used in financial management, review of a sample budget, and an introduction to the cashbook with exercises around distinguishing the different types of costs, the budget and the structure of the cashbook. Day three focused on documentation and financial reports as well as financial control systems and processes. The fourth day was reserved for practical exercises on all the books and the records management system.

Supplementary files have been included for the 4‐day training agenda (Data S2), exercises used to teach basic financial management concepts (Data S3), and exercises used to teach context‐specific financial recordkeeping and reporting concepts (Data S4).

### 2.3 Study design

Through project procedures to monitor the uptake and effectiveness of our financial management training program, we used pretraining and posttraining tests to (a) quantitatively assess the change in financial knowledge of individuals who participated in the trainings and (b) to determine the quality of the training implementation through participant feedback. Additionally, we conducted two rounds of in‐depth interviews (IDIs) at each site and assessed the completeness of financial records to discern the ability of GCs to practice what they had been taught.

### 2.4 Data collection

Each participant was given a short knowledge test at the very beginning of training and at the end of training after the completion of all learning modules. Each test included six questions worth a total of 10 points. For example, participants were given a specific cost item (eg, bed and cleaning supplies) and asked to say whether the item was a routine expenditure (every month) or periodic expenditure (not regular). Another question asked participants to calculate the bank balance and cash on hand given several revenue and expense transactions (Data S3). The posttest questionnaire had 12 additional questions, which assessed the quality of the training and trainers and solicited feedback on what the participants liked or did not like, which concepts could have been explained more clearly and suggestions for improvement. Seven of the questions were assessed on a 5‐point Likert‐like scale (strongly agree to strongly disagree), while the other five questions were open‐ended. All questionnaires were developed and administered in English; verbal translation in the local language was provided while the participants were answering the questionnaires. We have included the post‐test questionnaire as Data S5.

We conducted two rounds of IDI with income generating activity officers and GC members involved in the operations of the businesses in late 2017 and 1 year later. During the initial round of IDIs, the income generating activities had been operating between 1 and 7 months, the timing of which varied by site. During the second round of IDIs, the income generating activities had been operating between 12 and 18 months. One to two individuals were purposively selected at each site for participation in the interviews depending upon availability during the times of data collection. The IDI instruments elicited respondents' perspectives and opinions on the functioning of the income generating activities; whether they had the knowledge, skills, and resources to meet their responsibilities; the operations and finances of the income generating activities; the financial system overall; and the prospects for long‐term sustainability of the income generating activities and MWHs. Minimal demographic data were collected from each respondent and entered into SurveyCTO Collect Software (Dobility, Inc, Cambridge, MA) on tablets.

We extracted information from the cashbook of each site monthly using SurveyCTO Collect Software (Dobility, Inc, Cambridge, MA) on tablets from the opening of each site's income generating activity through July 2018, a period ranging from 9 to 16 months.

### 2.5 Analysis

Data from participants at the 2‐day and 4‐day trainings were analyzed separately. The knowledge questions from the pretest and posttest were each assigned points as described above. Points for correct answers were summed to create a total knowledge score out of 10 points; a higher score indicated better performance. Data from the pretest and posttests were compiled into an electronic database using Microsoft Excel and included respondent demographic data, position on the GC, knowledge pretest and posttest scores, and feedback responses on the trainer and training.

Descriptive statistics of the participants were calculated for the 2‐day training, the 4‐day training, and the combined sample. Chi‐squared tests were completed to test for differences in participant demographics between the two trainings. Two participants were dropped from the analysis because they lacked either a pretest or posttest score. The difference between the pretest and posttest scores was calculated as a “change score” and analyzed as a continuous variable. Pretest, posttest, and change scores are presented as means and standard deviations (SD) for each of the trainings. *t* tests were used to assess significant differences between the 2‐day and 4‐day training scores.

The change score was analyzed using paired *t*tests for the combined sample. Three multivariate linear regression models assessed pretest scores, posttest scores, and the change scores, controlling for length of training (2 days/4 days), participant education level, and sex. The pretest score was also controlled for in the posttest score and change score models. Position on the GC was not a control variable because of the collinearity of position and education. Categories with the most frequent response were used as the reference.^25^ Quantitative data were analyzed using the SAS v9.4 (Cary, NC) computing package. Results were considered statistically significant at the .05 level. Participant training feedback data, collected the day of the training, were manually coded using codes created *a priori* based on the questionnaire. The study team then conducted content analysis.^26^

IDIs were audio recorded, translated and transcribed into English, and coded in Nvivo v11 (QSR International, Doncaster, Australia) using a mixed inductive‐deductive approach to establish the codebook. The interviews were coded using content analysis.^26^ Demographic data were analyzed in SAS v9.4 (Cary, NC). Proportions or means and standard deviations are presented. Cashbook financial data for each site were reviewed at the end of the data collection period by a data analyst to determine whether the transactions were properly recorded, and their contents were summarized.

## 3 RESULTS

### 3.1 Training participant demographics

Of the 83 training participants in the combined sample, 52% (n = 43) were female. Most participants had some secondary education (71.1%) or more than secondary education (18.1%) (Table 2). There were no significant differences observed in sex, education level, or position held on the GC between the two trainings (2‐day vs. 4‐day).

**Table 2:** Demographic characteristics of participants combined and in the 2‐day and 4‐day financial trainings

		Total N = 83	2‐day Training (Pilot) N = 29	4‐day Training N = 54	*P* value
Sex, n (%)	Male	40 (48.2)	16 (55.2)	24 (44.4)	
	Female	43 (51.8)	13 (44.8)	30 (55.6)	
					.351
Education level, n (%)	Some primary	9 (10.8)	2 (6.9)	7 (13.0)	
	Some secondary	59 (71.1)	20 (69.0)	39 (72.2)	
	More than secondary	15 (18.1)	7 (24.1)	8 (14.8)	
					.455
Position on Governance Committee, n (%)	GC chairperson	9 (10.8)	3 (10.3)	6 (11.1)	
	Health center staff	8 (9.6)	2 (6.9)	6 (11.1)	
	Community health worker	9 (10.8)	4 (13.8)	5 (9.3)	
	IGA officer or IGA subcommittee member	29 (34.9)	10 (34.5)	19 (35.2)	
	MWH officer or MWH subcommittee member	15 (18.1)	5 (17.2)	10 (18.5)	
	Treasurer or Finance subcommittee	13 (15.7)	5 (17.2)	8 (14.8)	
					.977
Scores, mean (SD)^a^	Pre‐test	5.6 (2.6)	6.4 (1.8)	5.2 (2.9)	.027
	Post‐test	7.3 (2.2)	8.0 (1.8)	6.9 (2.3)	.027
	Change score	1.7 (2.1)	1.7 (2.2)	1.7 (2.0)	.950

Abbreviations: GC, governance committee; IGA, income generating activity; MWH, maternity waiting home.

a Two records were dropped for not having a pretest or posttest score (n = 81, 28, 53, respectively).

### 3.2 Training scores

The majority of participants had some baseline understanding of financial management (mean score 5.6, SD 2.6). Baseline knowledge was significantly lower in the 4‐day training group (mean score 5.2, SD 2.9) compared with the 2‐day training group (mean score 6.4, SD 1.8) (.027) (Table 2). Posttest scores were also significantly lower in the 4‐day training (6.9, SD 1.8) compared with the 2‐day training (8.0, SD 1.8) (.950). There was no statistically significantly difference in the change score between the 2‐day and 4‐day training (.95).

### 3.3 Change in knowledge

Table 3 presents the change scores stratified by demographic characteristics for the combined sample. We observed significant improvements after training as the mean change score was 1.7 (95% CI, 1.2‐2.2, *P* < .001). Participants with some secondary education (mean change score 1.9, 95% CI, 2.3‐2.4, *P* < .001) and those with more than secondary education (mean change score 1.5, 95% CI, 0.7‐2.3, .002) had statistically significant improvements in scores after the training.

**Table 3:** Mean change score (difference of pretest and posttest scores) for the combined 2‐day and 4‐day financial management trainings

		Mean Change Score	95% Confidence Interval	*P* value
Total		1.7	(1.2‐2.2)	<.001
Sex	Male	1.8	(1.1‐2.5)	<.001
	Female	1.6	(1.0‐2.2)	<.001
Position on the Governance Committee	GC chairperson	2.8	(1.2‐4.4)	.004
	Health center staff	0.8	(−0.1‐1.7)	.071
	Community health worker	2.1	(0.4‐3.8)	.021
	IGA officer or IGA subcommittee member	1.9	(1.1‐2.7)	<.001
	MWH officer or MWH subcommittee member	1.3	(0.2‐2.5)	.029
	Treasurer or Finance subcommittee	1.1	(−0.4‐2.7)	.132
Education	Some primary	0.8	(1.8‐4.8)	.347
	Some secondary	1.9	(2.3‐2.4)	<.001
	More than secondary	1.5	(0.7‐2.3)	.002

Abbreviations: GC, governance committee; IGA, income generating activity; MWH, maternity waiting home.

### 3.4 Models for pretest, posttest, and change score

Linear regression models for unadjusted and adjusted pretest, posttest, and change scores are shown in Table 4. First, after controlling for education and sex, there is no significant difference in pretest scores between the 2‐day and 4‐day training. However, after controlling for sex and type of training, participants with more than secondary education had a higher average pretest score of 3.8 (95% CI, 1.8‐5.9) points compared with those with some primary education (*P* < .001). Second, for the posttest score, participants with some secondary education scored 1.8 (95% CI, 0.6‐2.9) points higher than participants with some primary education (.003); those with more than secondary education had an average posttest score 2.9 (95% CI, 1.4‐4.4) points higher than those with some primary education (*P* < .001).

**Table 4:** Linear regression models estimating, pretest score, posttest score, and change score (the difference of pretest and post test scores) controlling for sex, position, and length of training

		Pretest				Posttest				Change score			
		Unadjusted β	*P* value	Adjusted β	*P* value	Unadjusted β	*P* value	Adjusted β	*P* value	Unadjusted β	*P* value	Adjusted β	*P* value
Sex	Male	−0.2 (−1.4, 0.9)	.658	−0.6 (−1.6, 0.5)	.283	−0.03 (−1.0, 1.0)	.957	−0.02 (−0.7, 0.7)	.957	0.2 (−0.7, 1.2)	.624	−0.02 (−0.7, 0.7)	.957
	Female	ref		ref		ref		ref		ref		ref	
Education	Some primary	ref		ref		ref		ref		ref		ref	
	Some secondary	1.3 (−0.4, 3.0)	.124	1.1 (−0.5, 2.8)	.180	2.4 (1.0, 3.7)	<.001	1.8 (0.6, 2.9)	.003	1.0 (−0.4, 2.5)	.480	1.8 (0.6, 2.9)	.003
	More than secondary	4.02 (2.0, 6.0)	<.001	3.8 (1.8, 5.9)	<.001	4.67 (3.0, 6.3)	<.001	2.9 (1.4, 4.4)	<.001	0.6 (−1.2, 2.4)	.162	2.9 (1.4, 4.4)	<.001
Length	2‐day training	1.2 (−0.01, 2.3)	.050	0.9 (−0.1, 2.1)	.088	1.1 (0.1, 2.1)	.027	0.5 (−0.3, 1.2)	.237	−0.03 (1.0, 0.9)	.950	0.5 (−0.3, 1.2)	.237
	4‐day training	ref		ref		ref		ref		ref		ref	
Pretest Score						0.5 (0.4, 0.7)	<.001	0.4 (0.2, 0.6)	<.001	−0.5 (−0.6, ‐0.3)	<.001	−0.6 (−0.7, −0.4)	<.001
				*R* ^2^	.23			*R* ^2^	.51			*R* ^2^	.45
				Model	<.001			Model	<.001			Model	<.001

Lastly, after controlling for other factors, the change scores for participants with some secondary education and more than secondary education were on average 1.8 (95% CI, 0.6‐2.9; .003) and 2.9 (95% CI, 1.4‐4.4; *P* < .001) points higher, respectively, than participants with some primary education.

### 3.5 Feedback on training quality and structure

Participants in the 2‐day training appreciated the interaction between the facilitators and participants felt that the level of the training was appropriate (ie, facilitators were “speaking on [their] level”) and liked the amount of time allocated for questions. The participants said they learned skills that could relate to their life and liked having a chance to practice and learn with the actual books they would be using for their respective income generating activity. They also appreciated learning about topics such as budgeting, community contributions, financial reports, and procurement. One male participant noted that “Staff and facilitators were very flexible and ready to answer questions and complaints.”

Many participants in the 2‐day training complained that the training was too short. They thought that the following topics could have been explained better: cashbook, capital vs expenditures, financial tracking/reporting, and budgeting. To improve the training course, participants suggested increasing the number of days, repeating the workshop, and holding follow‐up trainings. For example, a male finance subcommittee member said, “Repeat the course after six months so that we can present the challenges we identify after putting our knowledge into practice.” Participants also requested exchange visits and follow‐up mentorship. They observed that the implementation team works hard with the community, stating “By us following what we learned, and doing the work together, not alone, we can put together the required resources and be accountable more easily” (male, income generating activity subcommittee member). A female income generating activity subcommittee member stated her willingness to try to train others: “We will do the job, and we will teach others these topics.”

The open‐ended remarks at the end of the 4‐day training were generally positive. Participants commented on the friendliness of the facilitators and how they had learned new knowledge and skills including how to keep money, the purpose of various books, how to run a business, and manage a petty cash system. Participants praised the teaching methods used, commenting on the organization of materials and how the interactive sessions provided ample time for questions.

“The facilitators did not ‘teach' but involved us, getting us to participate fully.”—male, income generating activity officer.

“Please continue teaching in the same manner as how we were taught, from what the trainers knew and their experience. The teachings were presented well, and we really learned.”—female, governance committee secretary.

Participants of the 4‐day training suggested that language barriers were challenging. There were many local dialects being spoken at the same training, which made it difficult for some participants to fully comprehend the concepts. Participants also wanted a more thorough explanation of the cashbook and receipts and recommended that the trainers spend more time explaining financial management concepts. Although some participants of the 4‐day training also felt it was too short, there were fewer comments about the length of the training being insufficient compared to the 2‐day pilot training.

### 3.6 Interview respondent demographics

Twenty‐eight IDIs were conducted with income generating activty officers or sub‐committee members and a few treasurers (Table 5). Respondents were a mean of 39 years old; the majority were male, were farmers, and were on the GC overseeing the income generating activities rather than being the direct operators of the businesses. All respondents had attended some schooling, having completed a mean of approximately 10 years of schooling.

**Table 5:** Demographic characteristics of in‐depth interview respondents (n = 28)

Age (years), mean (SD)	39.1 (12.1)
Female, n (%)	9 (32.1)
Role for IGA, n (%)	
IGA officer	11 (33.3)
IGA subcommittee member	15 (53.6)
GC treasurer	2 (7.1)
Time in IGA role (years), mean (SD)	
Round 1 respondents	0.3 (0.3)
Round 2 respondents	1.0 (0.5)
Main occupation, n (%)	
Farmer	22 (78.5)
Businessman/woman	2 (7.1)
IGA Officer (only)	2 (7.1)
Other	2 (7.1)
Ever attended school, n (%)	28 (100)
Highest grade completed, mean (SD)^a^	9.7 (3.3)

Abbreviations: IGA, income generating activity; GC, governance committee.

a Missing 10.7% (n = 3) of the data for grade level but all completed some primary education.

### 3.7 Performance in practice

When asked about the functioning of the financial system, income generating activitiy officers and GC members expressed clear understanding of the principles of financial management covered during the trainings including accountability, transparency, and accurate recordkeeping. Even when the specific words of “accountability” and “transparency” were not used, the sites provided examples of these terms in action. For example, respondents frequently discussed the need to always track sales and income, the importance of reporting profits and the financial status of the income generating activity to the GC and to the larger community, the necessity of routinely banking profits to deter theft or misappropriation of funds, the requirement of receipts for documentation of procurements, and the regular supervision provided by the GC in reviewing the financial and sales books. Respondents were proud with their ability to master the financial, sales, and stock tracking books and procedures; many of the early successes mentioned in the interviews focused on this. There was a general appreciation for the system in place and a desire to adhere to it as closely as possible into the future. Example quotes of how the sites implemented the financial systems are included below:

“In the report we write the money that we have raised in that month, we also highlight the items that are selling well and those that are not selling well so that we make a decision whether to continue with the items that do not have much demand or to stop.”—agrodealership site.

“[The report] is good as it allows for the accountability. It allows the governance committee to know how the money was spent … .We write [down] the money that we requested, the money that we were given, and how we spent the money.”—grinding mill site

“The MWH can be a success if money is generated at the income generating activity. We need to buy things that are needed at the MWH and also at the income generating activity so that it continues running. If the money is misused, then when the goats are sick, you won't have money to buy drugs for the goats. So when you sell the [goat] manure, you buy drugs so that the goats can be injected...”—goat rearing site

“The books bring about transparency and how we are spending the money. They also show if we are making profits or not and they also show if at all the business is growing financially or not.”—agrodealership site

While most early challenges discussed during the interviews were minimal and were quickly resolved, a few sites experienced persistent challenges. At one agrodealership, the salesperson needed multiple reminders to record sales throughout the day. Other sites experienced challenges consistently banking income due to the distances of the nearest banks. Illustrutive quotes of these challenges are included below:

“Sometimes when closing the books, you find that the total is smaller than the money that you have. This is because there are many people around when you are selling, and sometimes you forget to write in the books as some people are in a hurry. And so I make mistakes when writing in the book.”—agrodealership site

“The challenges are that the bank is not in this area. it is in the main town, so depositing is a challenge because you have to find someone going to town or when someone is going for orders that is when they can deposit.”—agrodealership site

Goat rearing sites had the additional challenge of not gaining much experience with the financial system in practice during the course of the evaluation as few sales were made while the goat herds reproduced and grew. Because of this, the interviews at the goat rearing sites were noticeably spare on detail about the functioning of the financial system.

Although respondents were pleased with their ability to track the sales and finances of the income generating activities, sites consistently voiced concern over their ability to function when new GC members would be elected, since the new members would not receive training and mentorship from the implementing organization. Some sites discussed methods to mitigate this challenge by transitioning some new members onto the committee while still retaining some existing members so they could provide training and mentorship and all institutional knowledge would not be lost.

“Before the GC is dissolved, they need to teach those new people so that they have the knowledge on how to handle this business.”—agrodealership site

Qualitative interview results were corroborated by a review of the cashbooks at each site where the GC treasurer recorded financial transactions each month. The financial records at seven out of the ten sites were easy to understand. Revenue, expenses, and bank deposits and withdrawals were all recorded, and the transactions tallied, with only minor deviations. However, one agrodealership site had challenges with recordkeeping as nothing was recorded in the cashbook for a few months due to a long absence by the treasurer. Limited assessment could be made for the two goat rearing sites as there were few transactions during the evaluation period as the goat herd grew.

## 4 DISCUSSION

This study investigated the effectiveness of trainings designed to increase the skills of GC members in performing financial management tasks necessary to supervise a community‐owned health program. We found that both a 2‐day and 4‐day trainings significantly improved knowledge of financial concepts. There was little difference in participants' understanding of financial management materials as measured by a change test score between trainings of different lengths, although participant feedback suggests participants were more satisfied with the longer training.

The education level of participants played a critical role in their understanding financial management training materials, when controlling for other demographic variables. When implementing a community development intervention that requires capacity building in financial management, it is not always feasible to ensure community‐appointed officers have a required level of education. Therefore, it may be more appropriate to tailor the training to the participants by including special design elements (eg, basic math refresher exercises for those needing this support), splitting difficult topics into shorter modules, forming small groups to ensure a mix of educational levels, and introducing peer coaching.^27^ This could help ensure that community members with lower education levels are able to learn alongside those with higher education levels.^27^ Other noncognitive interventions might also be explored to help learners at all education levels, such as entertainment‐based education.^28^ However, tailoring trainings appropriately may require advanced knowledge of participant demographics and additional resources, which could be challenging for implementers under time and budget constraints.

In the Zambian setting, where the training model is more traditionally didactic, we found that applying a more participatory approach was well received. Based on early feedback, the training format was adapted to increase the number of exercises and examples to contextualize the topics in ways that would seem more relevant and therefore more meaningful to participants. For example, we increased time for active learning with examples and exercises drawn from family life such as a family budget and daily life transactions (Data S4 and S5). In addition, more group exercises were conducted to give ample time to the participants to share knowledge and help each other solve problems. Prior research has shown that learning is not only an individual activity but also a social event, and learning is enhanced when groups can interact and develop collective understanding.^29^ This style of training could enhance participant engagement and motivation.^30^ Group work can also help overcome shame or shyness.^31^ Some participants might be too shy or fearful of criticism to ask questions in the larger group. In a small group, this fear can be mitigated.

Moreover, both trainings were learner‐focused such that the agenda and curriculum were revised at the end of each day to match the needs of the participants to solidify learning. In addition, instructors deliberately identified participants who were struggling and offered individual attention and additional activities to better solidify concepts. Overall, more exercises and group works were conducted to allow participants to learn from the practical application of concepts and to maintain interest.^32^ Having multiple trainers also allowed for individualized attention and translations.

The multiple languages and dialects of the participants posed a challenge. While the 2‐day training was verbally translated into Chinyanja in real time by the local study team and worked well, the 4‐day training brought together participants from six sites from across the provinces with different dialects, which was challenging for the trainers and some participants. To adjust, the trainers enlisted health center staff as informal translators to help these participants. However, participants would have preferred a complete training in their own language. While this may be ideal, it requires sufficient time and budget. Additionally, many financial management concepts are challenging to explain in local languages, even for native speakers. However, if the local language is written and the participants are literate, the pretest and posttest instruments, handouts, and summary sheets of main concepts could be easily and relatively quickly translated into the required local languages, which may facilitate participant learning and reduce the amount of real‐time translations needing to occur.

Although it is widely agreed that the financial management is a critical skill, the logistical challenges of training participants at a community‐level with various education levels and in multiple languages are considerable. To do it properly, programs need to be well resourced, have materials and oration translated into multiple languages, and have sufficient time to train and mentor participants.^33^ Different strategies should be developed to match the needs of the participants. One approach could be to create a program that includes multiple ways to build skills: initial training, refresher training, follow‐up visits, and mentoring. Additionally, learning transfer could be facilitated by having the groups, which operated within the training continue to meet outside the course to routinely discuss successes and address challenges after the end of outside funding. This would allow the same small‐group supportive peer environment to continue after the end of implementing partner support. However, geographic spread of groups, transport costs, and language barriers need to be considered when assessing the feasibility and sustainability of this approach.

While the knowledge of participants on financial management concepts significantly increased after our project's training, substantial mentorship proved necessary to fully bridge the education‐practice divide. Two rounds of full‐day on‐site mentorship at each site and a multi‐day lessons learned workshop bringing all relevant GC members to a central location were held in the months after the trainings. Mentorship focused on accurate recordkeeping, adhering to financial controls such as timely banking of revenue, preparing financial reports, and being accountable to the community at large. While the project attempted to design a simplified financial system and manageable procedures for this specific intervention while adhering to international best practices, the financial books and systems still proved complicated for some GC members to master. Generally, during the months of follow‐up with the sites, GCs proved capable of implementing what they had learned during the trainings and mentorship visits. GCs were able to discuss the foundational principles of financial management, including transparency, accountability, and recordkeeping, and they also clearly documented revenue, expenses, and banking transactions. This shows they could take what they learned during training and “bridge the divide” by putting what they learned into practice.

## 5 LIMITATIONS

This study had several limitations. First, the study design did not allow us to quantitatively assess how participants performed on‐the‐job and link their job performance with their training scores. However, we were able to make qualitative assessments of the general capacity of GCs to implement what was learned during trainings and mentorship visits. It would be beneficial in the future to follow participants over time to see if increased knowledge is associated with better on‐the‐job performance and understand its contribution to the broader financial sustainability of the health program.^34^ Second, our pretest and posttest instrument only assessed knowledge acquisition. Other aspects of learning may also be important to the development of financial capacity, including changes in motivation, confidence, and perceived ability to perform tasks.,^28^, ^35^ Future studies should expand the assessment to consider these domains. Third, the instrument used to assess knowledge was specifically developed for these trainings. Because of the nature of the instrument used, the generalizability of these findings to a population outside of these trainings is unknown. Lastly, the qualitative IDIs were potentially subject to social desirability bias as respondents may have been saying what they believed the project‐associated data collectors wanted to hear. However, the likelihood that this substantially biased the results is minimal as the performance of the GCs was corroborated with review of the completeness of the cashbooks over a year of follow‐up.

## 6 ETHICAL CONSIDERATIONS

The Boston University Institutional Review Board (H‐35321; July 1, 2016) and the ERES Converge Institutional Review Board (Ref. No. 2016‐June‐023) in Zambia provided ethical approval for this study. Training participants provided consent upon agreeing to participate in the trainings. IDI respondents provided consent before beginning the interviews. Data collectors were trained in research ethics and qualitative interviewing techniques before each round of IDIs. Consenting and interviews were conducted in the language of the respondent's choosing.

## 7 CONCLUSION

Building financial management capacity is of increasing importance in low‐ and middle‐income countries to help local communities take ownership of development activities and manage resources. Our study showed an improvement in the financial management knowledge of the participants attending such trainings, with more highly educated community members gaining more knowledge. Future trainings may want to create special activities to ensure equal learning for participants of different education levels and ensure there is one common language for all training participants or host different trainings for those speaking different languages. In addition, trainings should allow adequate time for participants to gain confidence in using financial terms and applying skills. This type of adapted financial management training program could be used in similar settings to improve community oversight of resources intended to strengthen health systems or other community development initiatives.

## CONFLICT OF INTEREST

The authors have no conflicts of interest to declare.

## FUNDING INFORMATION

This program was developed and is being implemented in collaboration with MSD for Mothers, MSD's 10‐year, $500 million initiative to help create a world where no woman dies giving life. MSD for Mothers is an initiative of Merck & Co., Inc., Kenilworth, N.J., U.S.A. (MRK 1846‐06500.COL). The development of this article was additionally supported in part by the Bill & Melinda Gates Foundation (OPP1130329) and The ELMA Foundation (ELMA‐15‐F0017). The funders had no role in study design, data collection and analysis, decision to publish, or preparation of the manuscript. The content is solely the responsibility of the authors and does not reflect positions or policies of MSD, the Bill & Melinda Gates Foundation, or The ELMA Foundation.

## Supporting information

Data S1. Graphic depiction of the maternity waiting home financial system

Data S2. Financial Management Training Agenda

Data S3. Training exercises related to basic financial management concepts

Data S4. Training exercises related to financial record‐keeping

Data S5. Post‐test for financial management training
