# Supplementary figures and images for: Building financial management capacity for community ownership of development initiatives in rural Zambia
Source: Int J Health Plann Manage. 2019 May 23;35(1):36–51. doi: 10.1002/hpm.2810 (PMC7043374; doi:10.1002/hpm.2810)

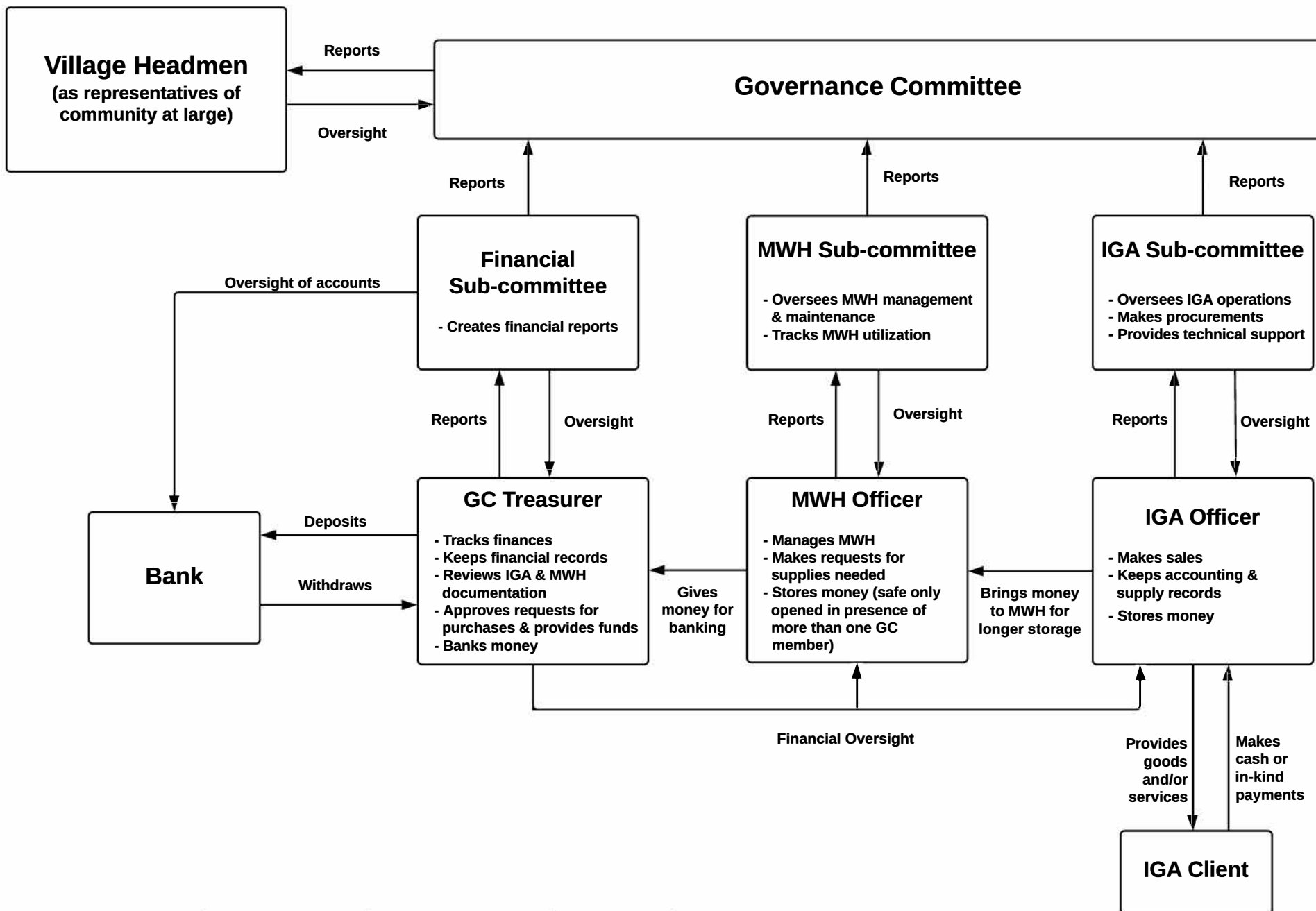

GC = governance committee; MWH = maternity waiting home, IGA = income generating activity

Supplement: Supplementary file 1 — Data S1. Graphic depiction of the maternity waiting home financial system [file HPM-35-36-s001.pdf]
