# Supplementary material for: Building financial management capacity for community ownership of development initiatives in rural Zambia
Source: Int J Health Plann Manage. 2019 May 23;35(1):36–51. doi: 10.1002/hpm.2810 (PMC7043374; doi:10.1002/hpm.2810)
Supplement: Supplementary file 2 — Data S2. Financial Management Training Agenda [file HPM-35-36-s002.docx]

**Financial Management Training Agenda**

**Day One**

9:00 – 9:30 **Registration and pre-test completion**

9:30 - 9:45 **Introductions, opening prayer**

9:45 - 10:00 **Welcoming remarks**

**Session 1** **Introduction to Financial Management Part 1**

10:00 - 10:45 **Exercise: Routine or Periodic Cost? Worksheet**

Handout – Routine or Periodic Cost

10:45 - 11:00 Health Break

11:00 - 1:00 **Introducing the Financial Procedure manual**

Working through the financial procedures manual with the participants

1:00 – 2:00 **Lunch**

2:00 – 4:30 **Small Group Activity: Play role: The procedure manual**

Working in small groups of 4, participants review the financial procedure manual and make a presentation to the community on the contents and importance of the procedure manual. Each group given 20 minutes to present.

4:30 – 5:00  **Day wrap up**

**Day Two**

**Session 2** **Introduction to Financial Management Part 2**

9:00-10:00 **Lecture on purpose of financial management and terms and definitions**

**Exercise: Routine or Periodic Cost? Worksheet**

Handout – Routine or Periodic Cost

10:00 – 10:30 **Small Group Activity: Mothers Shelter (MS) Budget Review**

Working in small groups for about 20 minutes, participants review a sample report (Handout) with estimated revenue and expenses for a MWH.

10:30 - 10:45 Health Break

10:45 – 11:30 Presentations of the group activity

**Session 3 Financial Transactions and the Cash Book**

11:30 - 1:00 **Lecture on Cashbook set up, how transactions are recorded**

1:00 – 2:00 **Lunch**

2:00 - 3:00 **Cashbook exercise**

Working in pairs, participants are given a list of financial transactions (Handouts).

3:00 - 4:00 **Cashbook report review, questions and answers**

4:00 – 4:30 **Day wrap up**

**Day Three**

**Session 4 Documentation and Financial Reports for IGAs**

9:00 – 9:45 Review of financial management documents

9:45 - 10:30 Exercise: Governance Committees create their document system

10:30 to 11:00 Health Break

11:00 to 11:30 Exercise: Governance Committees create their document system

11:45 – 1:00 Lecture on financial performance measures

1:00 to 2:00 Lunch

2:00 – 3:00 Role play on financial records and reporting

**Session 5 Control System and Policies (1 hr. 30 Minutes)**

3:00 – 4:30 Financial Policies and Control Systems

Lecture on controls, Review of policies

4:30 – 5:00 **Day wrap up**

**Day Four**

**Session 6 Practicals: System Design and Record Management System**

9:00 – 11:00 Creating Documents & Updating Records for IGAs

*Purchasing systems and documentation*

*Stock management system and documentation*

*Receiving cash and record keeping*

*Making payment and record keeping*

*Banking procedures and documentation*

*Report writing and presentations*

11:00 to 11:15 Health Break

11:15 to 1:00 Creating Documents & Updating Records for IGAs continues

1:00 to 2:00 Lunch

2:00 – 3:30 Creating Documents & Updating Records for IGAs continues

3:30 – 4:00 **Post-test and Evaluation**

4:00 – 5:00 **Closing Remarks**
