# Supplementary material for: Building financial management capacity for community ownership of development initiatives in rural Zambia
Source: Int J Health Plann Manage. 2019 May 23;35(1):36–51. doi: 10.1002/hpm.2810 (PMC7043374; doi:10.1002/hpm.2810)
Supplement: Supplementary file 3 — Data S3. Training exercises related to basic financial management concepts [file HPM-35-36-s003.docx]

**Exercise #1 – Routine or Periodic Cost?**

For each of the costs listed below, check the column for whether the cost is routine (meaning that it happens on a regular basis, monthly or almost monthly) or periodic (where you might have to replace something or pay for something once in a while, but it is not routine).

| **Cost Item** | **Routine?** | **Periodic?** |
| --- | --- | --- |
| Washing Soap |  |  |
| Cleaning supplies |  |  |
| Cupboard |  |  |
| Bed |  |  |
| Washing Dish |  |  |
| Mattress |  |  |
| Bed linens |  |  |
| Maintenance of furnishings (i.e. broken bed or cupboard) |  |  |
| Allowance for shelter staff (if paid) |  |  |

**Exercise #2 Maternity Waiting Home Annual Budgets**

Annual Budget for the period January 1, 2017 to December 31, 2017

| **Revenue** |  |
| --- | --- |
|  |  |
|  |  |
|  |  |
|  |  |
|  |  |
|  |  |
|  |  |
| Total |  |
|  |  |
|  |  |
| **Expenses** |  |
|  |  |
|  |  |
|  |  |
|  |  |
|  |  |
|  |  |
|  |  |
|  |  |
|  |  |
|  |  |
|  |  |
|  |  |
|  |  |
|  |  |
|  |  |
| Total |  |
|  |  |
| **Surplus/(Deficit)** |  |

**Exercise #3: Revenue or Expense?**

7/20 Opening cash balance of K500

7/25 K900 made from IGA sales

7/26 Withdrew cash out of bank, K300 on cheque number 001120

7/26 Machila the area MP, gave cash of K400 for MS donation

7/27 Purchased fuel for the IGA, K150

7/27 SMAG contributed cash of K150 to the mother shelter

7/27 Hired a maintenance worker to fix window, K124

7/28 Purchased washing soap, K29

7/28 Purchased grease for the IGA, K50

7/29 Hired a maintenance worker to fix IGA, K100

7/29 Bought engine oil for the IGA, K40

7/29 Bought a mosquito net, K40

7/29 Bought a ledger book, K20

7/30 Reimbursed GC members for transport, K60

7/30 Water bill, K25

7/31 Hired worker to unblock the toilet, K50
