# Supplementary material for: Building financial management capacity for community ownership of development initiatives in rural Zambia
Source: Int J Health Plann Manage. 2019 May 23;35(1):36–51. doi: 10.1002/hpm.2810 (PMC7043374; doi:10.1002/hpm.2810)
Supplement: Supplementary file 4 — Data S4. Training exercises related to financial record‐keeping [file HPM-35-36-s004.docx]

**Exercise #4: Fill out a cashbook**

Enter the below transactions into the cashbook in the appropriate rows and columns

**Sales from IGA activities**

05/01 Revenue from the agricultural sales K1900

07/01 Revenue from the grocery sales K1450

10/01 Revenue from the agricultural sales K1650

**Contributions**

17/01 The area MP, gave a cheque number 010104 of K1,800 for MWH donation

12/01 SMAG contributed cash of K150 to the MWH

13/01 Headmen contributed 20 bags of Maize

**Other Transactions**

24/01 Bought agricultural products, K1000

24/01 Hired a maintenance worker to fix window at Agrodealership, K100

24/01 Purchased Razor Blades, K50

25/01 Bought Biscuits, K40

25/01 Banked cash K3000 from IGA sales

26/01 Withdrew cash out of bank, 1200 by cheque

27/01 Hired a maintenance worker to fix window at MWH, K124

27/01 Replaced three beds at K1, 500 total by cheque number 032002

28/01 Purchased washing soap, K29

29/01 Bought a mosquito net, K40

29/01 Bought a ledger book, K20

30/01 Reimbursed GC members for transport, K60

31/01 Wages, K900 by cheque

**Exercise #5: Understand a petty cash system**

01/02 Dailes gave Management unit K2000 as petty cash

02/02 Richard requested for K75 to buy oil

14/02 Warredy requested for K500 to buy diesel/electricity/agro shop supplies

20/02 Bina Maluba brought receipt for maternal emergency K500 to the hospital

24/02 Jessy requested for soap for Mothers shelter, K100

24/02 Jessy returned K20 from soap

27/02 Makando requested for 2 door handles for Mothers shelter K150

28/02 Makando returned receipts for door handles

**Questions:**

1. How much is the FLOAT?
2. How much is total cash left?
3. How much is Top up?

**Exercise #6: Understanding stock control procedures**

**Agrodealership/Tuckshop Transactions**

01/01 Warredy received 10 bottles of Flea power

01/01 Ozzy received Needles 20 Adult and 10 infant

02/01 Ozzy received castration rub rings 6 packs

05/01 Warredy vitamins assorted for poultry 4 boxes, for cows 6 boxes, for goats 5 boxes

06/01 Litricia received seeds for cabbage, rape, Chinese, onion, tomato 10 packs each

01/01 Warredy received 10 packets of Finta Milk

01/01 Ozzy received Razor Blades 20 packets

02/01 Ozzy received 10 nappies, 20 soya chunks, 6 bottles of cooking oil, 15 small boom and 100 bubble gums

**Other Transactions**

06/01 Warredy sold 4 packets of Finta Milk

10.01 Ozzy sold 5 Adult needles, 3 infant needles, 2 castration rings, 1 box of cow vitamins and 1 box of vitamins for poultry

20/01 Warredy sold 1 pack of cabbage seed, 1 onion, 1 box of vitamins for goats and 1 pack castration ring rub

21/01 Ozzy sold 4 razor blade packs, 22 bubble gums, 2 nappies, 3 soya chunks and 1 bottle of cooking oil

02/01 Litricia received 15 bubble gums, 5 Adult needles and sold 5 small boom and 1 packet of milk

**Questions:**

1. Fill in stock card
2. What is your stock balance?
3. When do you reorder stock?

**Exercise #7: Fill out the sale journal**

Enter the below transactions into the sales journal in the appropriate rows and columns

**Agrodealership Transactions**

01/01 Warredy sold 10 bottles of Flea power @ K25 each

01/01 Ozzy received Needles 20 Adult and 10 infant @ K20 for Adult needles and K15 for infant needles

02/01 Ozzy sold castration rub rings 3 packs @ K5 per rub ring

05/01 Warredy sold vitamins assorted for poultry 4 boxes @ K 50, for cows 6 boxes @ K100 and for goats 5 boxes @ K60

06/01 Litricia sold seeds for cabbage, rape, Chinese, onion, tomato 10 packs @ K40 each

**Tuckshop Transactions**

01/01 Litricia sold 10 packets of Finta Milk @ K 20 each

01/01 Litricia sold Razor Blades 20 packets @ K 5 each

02/01 Litricia sold 10 nappies @ K20 each, 20 soya chunks @ K5 each, 6 bottles of cooking oil at K30 each, 15 small boom @ K15 and 100 bubble gums @ K1 each

**Exercise #8: Financial records and reporting to stakeholders**

It was a rainy day in Kacholola when the Chairperson for the mother shelter received a phone call from the secretary that the area MP had been to his home alleging that the Mother Shelter Governance Committee are misusing funds belong to the IGA. “*Bakamba bwanji?*”, exclaimed the chairperson. “Yes, he also said that the GC are using the hammer mill without paying” replied the Secretary. He also said that most people in the village are suspecting that the GC is not been transparent about the affairs of IGA and reported to the MP. The Chairperson said that such an allegation should be taken seriously and need to present facts to the community. The Chairperson further asked the secretary to call for a meeting to discuss this issue.

In the meeting, members resolved the following:

1. To call a meeting of all stakeholders in the community to explain the financial positions and control system that the GC have put in place
2. The treasurer was tasked to make available all the books and information that they use for all financial transactions.

**Required:**

1. Identify the stakeholders that will be invited to this meeting;
2. Prepare the Agenda for this community meeting;
3. Using the information available from the treasurer (see Exercise #4) present a financial report to the community;
4. Explain to the community the control system that the GC has put in place to safe guard the mother’s shelter resources.
