# Supplementary material for: Building financial management capacity for community ownership of development initiatives in rural Zambia
Source: Int J Health Plann Manage. 2019 May 23;35(1):36–51. doi: 10.1002/hpm.2810 (PMC7043374; doi:10.1002/hpm.2810)
Supplement: Supplementary file 5 — Data S5. Post‐test for financial management training [file HPM-35-36-s005.docx]

Name: ________________________ Health Facility Name: _________________________

Date: ______________________ Position: ____________________________

Gender: ________________________

This questionnaire is designed to measure your baseline understanding of concepts. We will use the information to help inform the training. We will repeat this test at the end.

**Circle the response that best describes your understanding of the term or concept.**

1. Financial management functions

(1) I have never heard of this

(2) I have heard of it but I don’t really know what it means

(3) I have some idea what this means, but it’s not too clear

(4) I have a clear idea what this means and can explain it

2. Budget

(1) I have never heard of this

(2) I have heard of it but I don’t really know what it means

(3) I have some idea what this means, but it’s not too clear

(4) I have a clear idea what this means and can explain it

3. Cost item or line item

(1) I have never heard of this

(2) I have heard of it but I don’t really know what it means

(3) I have some idea what this means, but it’s not too clear

(4) I have a clear idea what this means and can explain it

4. Expenditures and revenues

(1) I have never heard of this

(2) I have heard of it but I don’t really know what it means

(3) I have some idea what this means, but it’s not too clear

(4) I have a clear idea what this means and can explain it

5. Capital vs. recurrent expenditures

(1) I have never heard of this

(2) I have heard of it but I don’t really know what it means

(3) I have some idea what this means, but it’s not too clear

(4) I have a clear idea what this means and can explain it

6.Complete the table below by ticking against the type of expenditure that you think best describes the cost item

| **Cost item** | **Routine expenditure** | **Periodic expenditure** |
| --- | --- | --- |
| Bed |  |  |
| Cleaning supplies |  |  |
| Maintenance of furnishings |  |  |
| Mattress |  |  |

7. Financial transactions

(1) I have never heard of this

(2) I have heard of it but I don’t really know what it means

(3) I have some idea what this means, but it’s not too clear

(4) I have a clear idea what this means and can explain it

8. Procurement

(1) I have never heard of this

(2) I have heard of it but I don’t really know what it means

(3) I have some idea what this means, but it’s not too clear

(4) I have a clear idea what this means and can explain it

9.The purpose of financial management is:

- 1. To spend money
  2. To receive and spend money
  3. To receive, record, control and spend money

10.Financial management

- 1. Identifies areas for cost reduction
  2. Increases accountability
  3. Both and a and b

**Please choose the best response for the following questions.**

11. I can explain the role and responsibilities of MS Governance Committee (GC).

________COMPLETELY AGREE (5)

________ AGREE (4)

________ NEITHER AGREE NOR DISAGREE (3)

________ DISAGREE (2)

________COMPLETELY DISAGREE (1)

12. I can explain the purpose of financial management and why GC members should care about it.

________COMPLETELY AGREE (5)

________ AGREE (4)

________ NEITHER AGREE NOR DISAGREE (3)

________ DISAGREE (2)

________COMPLETELY DISAGREE (1)

13. I can describe the different types of revenue and categories of expenditures that the mothers’ shelter is likely to have.

________COMPLETELY AGREE (5)

________ AGREE (4)

________ NEITHER AGREE NOR DISAGREE (3)

________ DISAGREE (2)

________COMPLETELY DISAGREE (1)

14.Why is it important to keep track of finances?

……………………………………………………………………………………………………………………………………………………………………………………………………………………………………………………………………………………………………………………………………

15. I could review a cashbook and identify trends or detect possible errors / things that don’t look right.

________COMPLETELY AGREE (5)

________ AGREE (4)

________ NEITHER AGREE NOR DISAGREE (3)

________ DISAGREE (2)

________COMPLETELY DISAGREE (1)

16. If given a financial report about an income generating activity (IGA) would be able to assess whether the IGA is performing well or having financial difficulties.

________COMPLETELY AGREE (5)

________ AGREE (4)

________ NEITHER AGREE NOR DISAGREE (3)

________ DISAGREE (2)

________COMPLETELY DISAGREE (1)

17.A cash book is a list of all financial transactions. It includes:

1. Only revenue received
2. Payments made to buy things and revenue received
3. Only payments made to purchase things

18. Review the table below and calculate the balance brought forward for both money in the bank and money in the cash box.

|  |  |  | Bank | | Cash | |
| --- | --- | --- | --- | --- | --- | --- |
| Date | Description | Ref no | Receipts (+) | Payments (-) | Receipts (+) | Payments (-) |
| 1 Sept | Cash from IGA activities | 1 |  |  | 1,200.00 |  |
| 2 Sept | Donation from Health Facility | 4 |  |  | 2,000.00 |  |
| 3 Sept | Cash banked | 6 | 3,200.00 |  |  | 3,200.00 |
| 4 Sept | Cash withdrawn |  |  | 200 | 200.00 |  |
| 5 Sept | Food | 2 |  | 350 |  |  |
| 5 Sept | Stipends | 3 |  | 75 |  |  |
|  | Total for the Month |  | 3,200.00 | 625.00 | 3,400.00 | 3,250.00 |

Balance from previous month:

|  | **Bank** | **Cash** |
| --- | --- | --- |
| Total Receipts (+) |  |  |
| Total Payments (-) |  |  |
| **Balance going forward** |  |  |

**Please choose the best response to the following questions.**

19. I feel confident in my abilities to be a good Governing Board Member.

________COMPLETELY AGREE (5)

________ AGREE (4)

________ NEITHER AGREE NOR DISAGREE (3)

________ DISAGREE (2)

________COMPLETELY DISAGREE (1)

20. I consider myself mathematically weak.

________COMPLETELY AGREE (5)

________ AGREE (4)

________ NEITHER AGREE NOR DISAGREE (3)

________ DISAGREE (2)

________COMPLETELY DISAGREE (1)

21. I am a bit shy about asking questions when I don’t understand something.

________COMPLETELY AGREE (5)

________ AGREE (4)

________ NEITHER AGREE NOR DISAGREE (3)

________ DISAGREE (2)

________COMPLETELY DISAGREE (1)

22. It is more important to focus on results rather than on process details.

________COMPLETELY AGREE (5)

________ AGREE (4)

________ NEITHER AGREE NOR DISAGREE (3)

________ DISAGREE (2)

________COMPLETELY DISAGREE (1)
